# 1-(Hydroxy­meth­yl)pyrene

**DOI:** 10.1107/S1600536810002424

**Published:** 2010-01-23

**Authors:** Tobias Gruber, Wilhelm Seichter, Edwin Weber

**Affiliations:** a Institut für Organische Chemie, TU Bergakademie Freiberg, Leipziger Strasse 29, D-09596 Freiberg/Sachsen, Germany

## Abstract

The asymmetric unit of the title compound, C_17_H_12_O, contains two molecules, in which the fused aromatic ring systems are almost planar [maximum deviations = 0.0529 (9) and 0.0256 (9) Å]. In the crystal, aromatic π–π stacking inter­actions (perpendicular distance of centroids of about 3.4 Å) and strong O—H⋯O hydrogen bonds result in a helical arrangement of pyrenyl dimers.

## Related literature

For the solid–state structures of pyrenes, see: Robertson & White (1947 ▶); Camerman & Trotter (1965 ▶); Allmann (1970 ▶); Hazell *et al.* (1972 ▶); Kai *et al.* (1978 ▶); Frampton *et al.* (2000 ▶). For the synthesis and structures of pyrene derivatives, see: Steward (1960 ▶); Gruber *et al.* (2006 ▶, 2008 ▶, 2009 ▶). For the use of pyrenes in fluorescence sensors, see: Bren (2001 ▶).
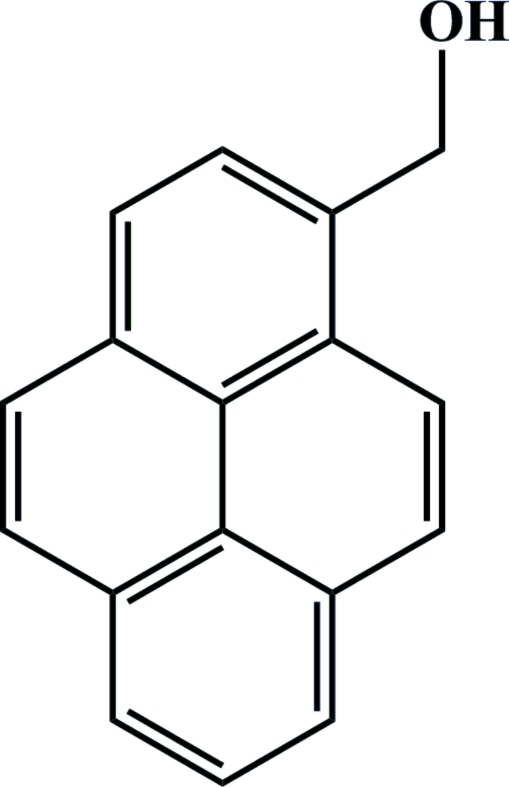


## Experimental

### 

#### Crystal data

C_17_H_12_O
*M* *_r_* = 232.27
Monoclinic, 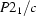

*a* = 19.9182 (6) Å
*b* = 8.8880 (3) Å
*c* = 13.0882 (4) Å
β = 91.719 (2)°
*V* = 2316.00 (13) Å^3^
*Z* = 8
Mo *K*α radiation
μ = 0.08 mm^−1^
*T* = 153 K
0.59 × 0.29 × 0.12 mm

#### Data collection

Bruker APEXII CCD area-detector diffractometer
29632 measured reflections
5051 independent reflections
3801 reflections with *I* > 2σ(*I*)
*R*_int_ = 0.026

#### Refinement

*R*[*F* ^2^ > 2σ(*F* ^2^)] = 0.038
*wR*(*F* ^2^) = 0.111
*S* = 1.06
5051 reflections
327 parameters
H-atom parameters constrained
Δρ_max_ = 0.21 e Å^−3^
Δρ_min_ = −0.18 e Å^−3^

### 

Data collection: *APEX2* (Bruker, 2004 ▶); cell refinement: *SAINT* (Bruker, 2004 ▶); data reduction: *SAINT*; program(s) used to solve structure: *SHELXS97* (Sheldrick, 2008 ▶); program(s) used to refine structure: *SHELXL97* (Sheldrick, 2008 ▶); molecular graphics: *ORTEP-3* (Farrugia, 1997 ▶); software used to prepare material for publication: *SHELXTL* (Sheldrick, 2008 ▶).

## Supplementary Material

Crystal structure: contains datablocks global, I. DOI: 10.1107/S1600536810002424/rk2186sup1.cif

Structure factors: contains datablocks I. DOI: 10.1107/S1600536810002424/rk2186Isup2.hkl

Additional supplementary materials:  crystallographic information; 3D view; checkCIF report

## Figures and Tables

**Table 1:** Hydrogen-bond geometry (Å, °)

*D*—H⋯*A*	*D*—H	H⋯*A*	*D*⋯*A*	*D*—H⋯*A*
O1—H1⋯O1*A*^i^	0.84	1.87	2.6972 (12)	167
O1*A*—H1*A*⋯O1^ii^	0.84	1.89	2.7163 (12)	167

Symmetry codes: (i) 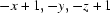; (ii) 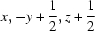.

**Fig. 1.**
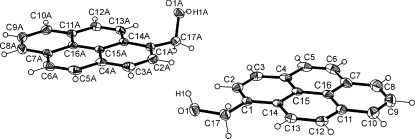
Molecular structure of the title compound with the atom numbering scheme. Displacement ellipsoids are drawn at 30% probability level. H atoms are presented as a small cyrcles of arbitrary radius.

**Fig. 2.**
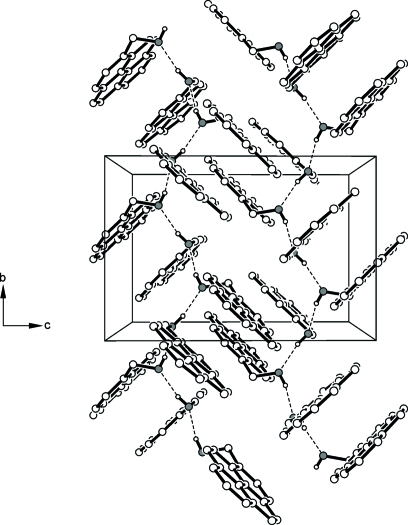
Packing diagram of the title compound, viewed down the a axis. Hydrogen atoms not involved in hydrogen bonding have been omitted.

## supplementary crystallographic information

### Comment

Owing to their electronic, optical and geometric properties, monofunctionalized pyrenes, attachable to a receptor platform, are of special interest for fluorescent sensor development (Bren, 2001). In this respect, 1–(hydroxymethyl)pyrene was prepared as part of our studies on the solid state structure of fluorogenic calixarenes with possible analytical applications (Gruber *et al.*, 2008; Gruber *et al.*, 2009).

Being composed of a plane aromatic region and a methylene bridged hydroxy group, the hybrid nature of the title compound is striking. The pyrene moiety alone shows no significant deviations of bond lengths and angles compared with those of the unsubstituted analogue (Robertson & White, 1947; Camerman & Trotter, 1965; Allmann, 1970; Hazell *et al.*, 1972; Kai *et al.*, 1978), and is almost planar. The largest deviation from the mean plane through the carbon framework of the pyrene unit is observed for atoms C2 [0.0529 (9)Å] and C1A [0.0256 (9)Å], respectively. Similiar to the unsubstituted parent substance, the pyrene moities of two molecules of 1–(hydroxymethyl)pyrene are forming a slightly displaced face–to–face dimer with an average distance of the aromatic units of about 3.4Å, though the latter are not arranged entirely coplanar [2.43 (3)°]. Additionally, within the dimer a strong hydrogen bond involving the two hydroxy groups can be observed [*d*(O···O) = 2.6972 (12)Å]. Worth mentoining is the varying conformation of the hydroxymethyl residue in both molecules of the asymmetrtic unit. In molecule 1, a nearly coplanar arrangement with regard to the aromatic plane can be observed [C2–C1–C17–O1 = 3.46 (15)°], whereas in molecule 2 the same torsion angle of 116.54 (12)° is adopted (Fig. 1). These findings are explained by the sterical demands of a strong hydrogen bond between two hydroxy groups [*d*(O···O) = 2.7163 (12)Å], which links the pyrene dimers mentioned above in a helical manner in the direction of the crystallographic *b* axis. Considering the packing, two of these helices, each in the opposite direction, are connected by edge–to–face interactions of the pyrenyl groups as shown in Fig. 2.

### Experimental

The title compound was synthesized from commercially available pyrene–1–carbaldehyde, which was reduced with sodium borohydride in boiling methanol, following an analogous procedure described for the reduction of anthracene–9–carbaldehyde (Steward, 1960; Gruber *et al.*, 2006). Colourless plates (m.p. 393–394 K) of the solvent–free 1–(hydroxymethyl)pyrene suitable for X–ray diffraction were obtained by recrystallization from *n*–hexane/dichloromethane (1:2).

### Refinement

The H atoms were positioned geometrically and allowed to ride on their parent atoms, with O—H = 0.84Å, C—H = 0.95–0.99Å and *U*_iso_ = 1.2–1.5 *U*_eq_(parent atom).

### Crystal data

**Table:**

C_17_H_12_O	*F*(000) = 976
*M**_r_* = 232.27	*D*_x_ = 1.332 Mg m^−^^3^
Monoclinic, *P*2_1_/*c*	Melting point: 393 K
Hall symbol: -P 2ybc	Mo *K*α radiation, λ = 0.71073 Å
*a* = 19.9182 (6) Å	Cell parameters from 8740 reflections
*b* = 8.8880 (3) Å	θ = 2.5–32.3°
*c* = 13.0882 (4) Å	µ = 0.08 mm^−^^1^
β = 91.719 (2)°	*T* = 153 K
*V* = 2316.00 (13) Å^3^	Plate, colourless
*Z* = 8	0.59 × 0.29 × 0.12 mm

### Data collection

**Table:**

Bruker APEXII CCD area-detector diffractometer	3801 reflections with *I* > 2σ(*I*)
Radiation source: fine–focus sealed tube	*R*_int_ = 0.026
graphite	θ_max_ = 27.0°, θ_min_ = 3.0°
φ and ω scans	*h* = −25→25
29632 measured reflections	*k* = −11→10
5051 independent reflections	*l* = −16→11

### Refinement

**Table:**

Refinement on *F*^2^	Primary atom site location: structure-invariant direct methods
Least-squares matrix: full	Secondary atom site location: difference Fourier map
*R*[*F*^2^ > 2σ(*F*^2^)] = 0.038	Hydrogen site location: inferred from neighbouring sites
*wR*(*F*^2^) = 0.111	H-atom parameters constrained
*S* = 1.06	*w* = 1/[σ^2^(*F*_o_^2^) + (0.0591*P*)^2^ + 0.2704*P*] where *P* = (*F*_o_^2^ + 2*F*_c_^2^)/3
5051 reflections	(Δ/σ)_max_ < 0.001
327 parameters	Δρ_max_ = 0.21 e Å^−^^3^
0 restraints	Δρ_min_ = −0.18 e Å^−^^3^

### Special details

**Table:**

Geometry. All s.u.'s (except the s.u. in the dihedral angle between two l.s. planes) are estimated using the full covariance matrix. The cell s.u.'s are taken into account individually in the estimation of s.u.'s in distances, angles and torsion angles; correlations between s.u.'s in cell parameters are only used when they are defined by crystal symmetry. An approximate (isotropic) treatment of cell s.u.'s is used for estimating s.u.'s involving l.s. planes.
Refinement. Refinement of *F*^2^ against ALL reflections. The weighted *R*–factor *wR* and goodness of fit *S* are based on *F*^2^, conventional *R*–factors *R* are based on *F*, with *F* set to zero for negative *F*^2^. The threshold expression of *F*^2^ > σ(*F*^2^) is used only for calculating *R*–factors(gt) *etc*. and is not relevant to the choice of reflections for refinement. *R*–factors based on *F*^2^ are statistically about twice as large as those based on *F*, and *R*–factors based on ALL data will be even larger.

### Fractional atomic coordinates and isotropic or equivalent isotropic displacement parameters (Å^2^)

**Table:**

	*x*	*y*	*z*	*U*_iso_*/*U*_eq_	
O1	0.53989 (4)	−0.02633 (10)	0.21742 (7)	0.0412 (2)	
H1	0.5295	−0.0899	0.2617	0.062*	
C1	0.65534 (6)	0.05248 (12)	0.26246 (8)	0.0262 (2)	
C2	0.63001 (6)	0.15072 (13)	0.33402 (8)	0.0316 (3)	
H2	0.5828	0.1582	0.3409	0.038*	
C3	0.67218 (6)	0.23825 (13)	0.39567 (8)	0.0336 (3)	
H3	0.6534	0.3036	0.4447	0.040*	
C4	0.74142 (6)	0.23177 (12)	0.38682 (8)	0.0296 (3)	
C5	0.78651 (7)	0.32180 (13)	0.44853 (9)	0.0392 (3)	
H5	0.7688	0.3864	0.4989	0.047*	
C6	0.85288 (7)	0.31708 (14)	0.43697 (10)	0.0444 (3)	
H6	0.8812	0.3781	0.4794	0.053*	
C7	0.88234 (7)	0.22197 (14)	0.36198 (10)	0.0388 (3)	
C8	0.95132 (7)	0.21558 (18)	0.34778 (12)	0.0540 (4)	
H8	0.9807	0.2747	0.3899	0.065*	
C9	0.97759 (7)	0.1249 (2)	0.27363 (13)	0.0606 (5)	
H9	1.0247	0.1236	0.2645	0.073*	
C10	0.93612 (7)	0.03560 (18)	0.21215 (11)	0.0494 (4)	
H10	0.9550	−0.0256	0.1609	0.059*	
C11	0.86674 (6)	0.03471 (14)	0.22482 (9)	0.0334 (3)	
C12	0.82208 (6)	−0.05924 (13)	0.16581 (9)	0.0334 (3)	
H12	0.8400	−0.1256	0.1167	0.040*	
C13	0.75524 (6)	−0.05603 (12)	0.17800 (8)	0.0282 (3)	
H13	0.7272	−0.1204	0.1374	0.034*	
C14	0.72512 (5)	0.04227 (11)	0.25087 (7)	0.0236 (2)	
C15	0.76866 (6)	0.13372 (11)	0.31289 (8)	0.0250 (2)	
C16	0.83916 (6)	0.12977 (12)	0.29987 (8)	0.0296 (3)	
C17	0.60896 (6)	−0.04252 (14)	0.19512 (9)	0.0335 (3)	
H17A	0.6153	−0.0146	0.1228	0.040*	
H17B	0.6218	−0.1496	0.2032	0.040*	
O1A	0.48228 (4)	0.26209 (9)	0.65830 (6)	0.0368 (2)	
H1A	0.4998	0.3385	0.6859	0.055*	
C1A	0.41705 (5)	0.19195 (13)	0.50620 (8)	0.0286 (3)	
C2A	0.43258 (6)	0.08575 (14)	0.43237 (9)	0.0343 (3)	
H2A	0.4781	0.0724	0.4148	0.041*	
C3A	0.38356 (6)	−0.00083 (14)	0.38391 (8)	0.0337 (3)	
H3A	0.3959	−0.0728	0.3342	0.040*	
C4A	0.31636 (6)	0.01636 (12)	0.40716 (8)	0.0277 (2)	
C5A	0.26394 (7)	−0.06959 (13)	0.35757 (8)	0.0341 (3)	
H5A	0.2754	−0.1425	0.3080	0.041*	
C6A	0.19914 (7)	−0.04986 (13)	0.37933 (9)	0.0360 (3)	
H6A	0.1658	−0.1081	0.3442	0.043*	
C7A	0.17913 (6)	0.05748 (13)	0.45456 (8)	0.0308 (3)	
C8A	0.11218 (6)	0.08017 (15)	0.47954 (10)	0.0397 (3)	
H8A	0.0779	0.0234	0.4455	0.048*	
C9A	0.09507 (6)	0.18397 (16)	0.55308 (10)	0.0426 (3)	
H9A	0.0492	0.1978	0.5688	0.051*	
C10A	0.14416 (6)	0.26767 (15)	0.60382 (9)	0.0371 (3)	
H10A	0.1317	0.3383	0.6543	0.045*	
C11A	0.21194 (6)	0.24959 (12)	0.58179 (8)	0.0281 (3)	
C12A	0.26408 (6)	0.33496 (13)	0.63165 (8)	0.0305 (3)	
H12A	0.2526	0.4059	0.6826	0.037*	
C13A	0.32911 (6)	0.31781 (12)	0.60855 (8)	0.0286 (3)	
H13A	0.3622	0.3777	0.6429	0.034*	
C14A	0.34959 (5)	0.21105 (12)	0.53313 (8)	0.0245 (2)	
C15A	0.29880 (5)	0.12329 (11)	0.48278 (7)	0.0232 (2)	
C16A	0.23004 (5)	0.14309 (12)	0.50639 (8)	0.0250 (2)	
C17A	0.47224 (6)	0.28837 (15)	0.55113 (9)	0.0370 (3)	
H17C	0.5145	0.2669	0.5159	0.044*	
H17D	0.4609	0.3956	0.5397	0.044*	

### Atomic displacement parameters (Å^2^)

**Table:**

	*U*^11^	*U*^22^	*U*^33^	*U*^12^	*U*^13^	*U*^23^
O1	0.0302 (5)	0.0379 (5)	0.0551 (6)	−0.0008 (4)	−0.0045 (4)	0.0141 (4)
C1	0.0342 (6)	0.0209 (5)	0.0234 (5)	0.0014 (5)	−0.0018 (4)	0.0054 (4)
C2	0.0356 (6)	0.0297 (6)	0.0297 (6)	0.0062 (5)	0.0051 (5)	0.0060 (5)
C3	0.0529 (8)	0.0252 (6)	0.0231 (6)	0.0098 (5)	0.0058 (5)	−0.0004 (5)
C4	0.0489 (7)	0.0184 (6)	0.0211 (5)	0.0018 (5)	−0.0035 (5)	0.0024 (4)
C5	0.0675 (9)	0.0225 (6)	0.0269 (6)	−0.0012 (6)	−0.0102 (6)	−0.0014 (5)
C6	0.0669 (10)	0.0280 (7)	0.0369 (7)	−0.0142 (6)	−0.0201 (6)	0.0039 (5)
C7	0.0451 (7)	0.0328 (7)	0.0377 (7)	−0.0109 (6)	−0.0120 (5)	0.0143 (5)
C8	0.0460 (8)	0.0608 (10)	0.0545 (9)	−0.0216 (7)	−0.0128 (7)	0.0200 (8)
C9	0.0329 (7)	0.0859 (12)	0.0627 (10)	−0.0102 (8)	−0.0017 (7)	0.0288 (9)
C10	0.0401 (8)	0.0603 (9)	0.0481 (8)	0.0083 (7)	0.0065 (6)	0.0171 (7)
C11	0.0344 (6)	0.0344 (7)	0.0316 (6)	0.0045 (5)	0.0012 (5)	0.0120 (5)
C12	0.0445 (7)	0.0297 (6)	0.0262 (6)	0.0108 (5)	0.0044 (5)	0.0020 (5)
C13	0.0392 (7)	0.0224 (6)	0.0229 (5)	0.0024 (5)	−0.0027 (4)	−0.0003 (4)
C14	0.0342 (6)	0.0176 (5)	0.0188 (5)	0.0018 (4)	−0.0016 (4)	0.0033 (4)
C15	0.0371 (6)	0.0177 (5)	0.0202 (5)	0.0015 (4)	−0.0030 (4)	0.0050 (4)
C16	0.0374 (7)	0.0244 (6)	0.0268 (6)	−0.0026 (5)	−0.0049 (5)	0.0102 (4)
C17	0.0322 (6)	0.0327 (7)	0.0351 (6)	−0.0021 (5)	−0.0036 (5)	0.0026 (5)
O1A	0.0400 (5)	0.0354 (5)	0.0342 (5)	−0.0092 (4)	−0.0105 (3)	0.0040 (4)
C1A	0.0319 (6)	0.0281 (6)	0.0257 (6)	−0.0012 (5)	−0.0023 (4)	0.0072 (4)
C2A	0.0324 (6)	0.0411 (7)	0.0297 (6)	0.0056 (5)	0.0043 (5)	0.0065 (5)
C3A	0.0468 (7)	0.0307 (6)	0.0236 (6)	0.0090 (5)	0.0033 (5)	−0.0016 (5)
C4A	0.0420 (7)	0.0206 (5)	0.0202 (5)	0.0009 (5)	−0.0029 (4)	0.0029 (4)
C5A	0.0568 (8)	0.0233 (6)	0.0219 (6)	−0.0028 (5)	−0.0059 (5)	−0.0013 (4)
C6A	0.0508 (8)	0.0282 (6)	0.0281 (6)	−0.0129 (6)	−0.0143 (5)	0.0037 (5)
C7A	0.0358 (7)	0.0281 (6)	0.0279 (6)	−0.0056 (5)	−0.0083 (5)	0.0104 (5)
C8A	0.0346 (7)	0.0413 (7)	0.0425 (7)	−0.0083 (6)	−0.0101 (5)	0.0161 (6)
C9A	0.0292 (6)	0.0506 (8)	0.0481 (8)	0.0017 (6)	0.0014 (5)	0.0206 (7)
C10A	0.0387 (7)	0.0384 (7)	0.0345 (6)	0.0106 (5)	0.0069 (5)	0.0104 (5)
C11A	0.0341 (6)	0.0257 (6)	0.0244 (5)	0.0044 (5)	−0.0002 (4)	0.0070 (4)
C12A	0.0430 (7)	0.0244 (6)	0.0239 (5)	0.0067 (5)	−0.0017 (5)	−0.0022 (4)
C13A	0.0383 (6)	0.0214 (6)	0.0257 (6)	−0.0009 (5)	−0.0080 (4)	−0.0012 (4)
C14A	0.0315 (6)	0.0197 (5)	0.0220 (5)	0.0001 (4)	−0.0032 (4)	0.0042 (4)
C15A	0.0330 (6)	0.0174 (5)	0.0190 (5)	0.0002 (4)	−0.0033 (4)	0.0036 (4)
C16A	0.0321 (6)	0.0205 (5)	0.0221 (5)	−0.0002 (4)	−0.0043 (4)	0.0072 (4)
C17A	0.0349 (6)	0.0411 (7)	0.0349 (7)	−0.0081 (6)	−0.0028 (5)	0.0093 (5)

### Geometric parameters (Å, °)

**Table:**

O1—C17	1.4222 (14)	O1A—C17A	1.4301 (14)
O1—H1	0.8400	O1A—H1A	0.8400
C1—C2	1.3865 (15)	C1A—C2A	1.3922 (17)
C1—C14	1.4056 (15)	C1A—C14A	1.4094 (15)
C1—C17	1.5146 (15)	C1A—C17A	1.5000 (16)
C2—C3	1.3860 (17)	C2A—C3A	1.3824 (17)
C2—H2	0.9500	C2A—H2A	0.9500
C3—C4	1.3887 (17)	C3A—C4A	1.3900 (16)
C3—H3	0.9500	C3A—H3A	0.9500
C4—C15	1.4218 (15)	C4A—C15A	1.4232 (15)
C4—C5	1.4338 (16)	C4A—C5A	1.4334 (16)
C5—C6	1.3356 (19)	C5A—C6A	1.3415 (18)
C5—H5	0.9500	C5A—H5A	0.9500
C6—C7	1.434 (2)	C6A—C7A	1.4360 (17)
C6—H6	0.9500	C6A—H6A	0.9500
C7—C8	1.393 (2)	C7A—C8A	1.3971 (17)
C7—C16	1.4247 (16)	C7A—C16A	1.4233 (15)
C8—C9	1.377 (2)	C8A—C9A	1.383 (2)
C8—H8	0.9500	C8A—H8A	0.9500
C9—C10	1.386 (2)	C9A—C10A	1.3825 (19)
C9—H9	0.9500	C9A—H9A	0.9500
C10—C11	1.3969 (18)	C10A—C11A	1.3983 (16)
C10—H10	0.9500	C10A—H10A	0.9500
C11—C16	1.4187 (17)	C11A—C16A	1.4216 (15)
C11—C12	1.4295 (17)	C11A—C12A	1.4281 (16)
C12—C13	1.3459 (16)	C12A—C13A	1.3476 (16)
C12—H12	0.9500	C12A—H12A	0.9500
C13—C14	1.4377 (15)	C13A—C14A	1.4371 (15)
C13—H13	0.9500	C13A—H13A	0.9500
C14—C15	1.4246 (14)	C14A—C15A	1.4235 (14)
C15—C16	1.4201 (16)	C15A—C16A	1.4241 (15)
C17—H17A	0.9900	C17A—H17C	0.9900
C17—H17B	0.9900	C17A—H17D	0.9900
			
C17—O1—H1	109.5	C17A—O1A—H1A	109.5
C2—C1—C14	119.64 (10)	C2A—C1A—C14A	119.25 (10)
C2—C1—C17	121.08 (10)	C2A—C1A—C17A	118.94 (11)
C14—C1—C17	119.28 (10)	C14A—C1A—C17A	121.74 (11)
C3—C2—C1	121.36 (11)	C3A—C2A—C1A	121.82 (11)
C3—C2—H2	119.3	C3A—C2A—H2A	119.1
C1—C2—H2	119.3	C1A—C2A—H2A	119.1
C2—C3—C4	120.94 (10)	C2A—C3A—C4A	120.66 (11)
C2—C3—H3	119.5	C2A—C3A—H3A	119.7
C4—C3—H3	119.5	C4A—C3A—H3A	119.7
C3—C4—C15	118.84 (10)	C3A—C4A—C15A	118.95 (10)
C3—C4—C5	122.47 (11)	C3A—C4A—C5A	122.37 (10)
C15—C4—C5	118.69 (11)	C15A—C4A—C5A	118.68 (10)
C6—C5—C4	121.60 (12)	C6A—C5A—C4A	121.78 (11)
C6—C5—H5	119.2	C6A—C5A—H5A	119.1
C4—C5—H5	119.2	C4A—C5A—H5A	119.1
C5—C6—C7	121.51 (11)	C5A—C6A—C7A	121.40 (10)
C5—C6—H6	119.2	C5A—C6A—H6A	119.3
C7—C6—H6	119.2	C7A—C6A—H6A	119.3
C8—C7—C16	118.77 (13)	C8A—C7A—C16A	118.86 (11)
C8—C7—C6	122.73 (12)	C8A—C7A—C6A	122.87 (11)
C16—C7—C6	118.50 (12)	C16A—C7A—C6A	118.27 (11)
C9—C8—C7	121.00 (13)	C9A—C8A—C7A	121.06 (12)
C9—C8—H8	119.5	C9A—C8A—H8A	119.5
C7—C8—H8	119.5	C7A—C8A—H8A	119.5
C8—C9—C10	120.81 (13)	C10A—C9A—C8A	120.51 (12)
C8—C9—H9	119.6	C10A—C9A—H9A	119.7
C10—C9—H9	119.6	C8A—C9A—H9A	119.7
C9—C10—C11	120.59 (14)	C9A—C10A—C11A	120.82 (12)
C9—C10—H10	119.7	C9A—C10A—H10A	119.6
C11—C10—H10	119.7	C11A—C10A—H10A	119.6
C10—C11—C16	118.93 (12)	C10A—C11A—C16A	119.09 (11)
C10—C11—C12	122.74 (12)	C10A—C11A—C12A	122.55 (11)
C16—C11—C12	118.32 (10)	C16A—C11A—C12A	118.36 (10)
C13—C12—C11	121.68 (11)	C13A—C12A—C11A	121.88 (10)
C13—C12—H12	119.2	C13A—C12A—H12A	119.1
C11—C12—H12	119.2	C11A—C12A—H12A	119.1
C12—C13—C14	121.70 (10)	C12A—C13A—C14A	121.56 (10)
C12—C13—H13	119.2	C12A—C13A—H13A	119.2
C14—C13—H13	119.2	C14A—C13A—H13A	119.2
C1—C14—C15	119.25 (9)	C1A—C14A—C15A	119.19 (10)
C1—C14—C13	122.99 (10)	C1A—C14A—C13A	122.93 (10)
C15—C14—C13	117.76 (10)	C15A—C14A—C13A	117.87 (10)
C16—C15—C4	119.72 (10)	C4A—C15A—C14A	120.12 (10)
C16—C15—C14	120.31 (10)	C4A—C15A—C16A	119.43 (10)
C4—C15—C14	119.95 (10)	C14A—C15A—C16A	120.44 (9)
C11—C16—C15	120.17 (10)	C11A—C16A—C7A	119.67 (10)
C11—C16—C7	119.86 (11)	C11A—C16A—C15A	119.90 (10)
C15—C16—C7	119.96 (11)	C7A—C16A—C15A	120.43 (10)
O1—C17—C1	113.64 (10)	O1A—C17A—C1A	111.75 (9)
O1—C17—H17A	108.8	O1A—C17A—H17C	109.3
C1—C17—H17A	108.8	C1A—C17A—H17C	109.3
O1—C17—H17B	108.8	O1A—C17A—H17D	109.3
C1—C17—H17B	108.8	C1A—C17A—H17D	109.3
H17A—C17—H17B	107.7	H17C—C17A—H17D	107.9
			
C14—C1—C2—C3	−0.83 (16)	C14A—C1A—C2A—C3A	0.59 (17)
C17—C1—C2—C3	−179.82 (10)	C17A—C1A—C2A—C3A	−176.38 (10)
C1—C2—C3—C4	0.83 (17)	C1A—C2A—C3A—C4A	0.44 (18)
C2—C3—C4—C15	0.19 (16)	C2A—C3A—C4A—C15A	−0.83 (16)
C2—C3—C4—C5	179.32 (10)	C2A—C3A—C4A—C5A	179.05 (10)
C3—C4—C5—C6	−178.12 (11)	C3A—C4A—C5A—C6A	−178.71 (11)
C15—C4—C5—C6	1.01 (17)	C15A—C4A—C5A—C6A	1.17 (16)
C4—C5—C6—C7	0.19 (18)	C4A—C5A—C6A—C7A	−0.76 (17)
C5—C6—C7—C8	179.54 (12)	C5A—C6A—C7A—C8A	−179.58 (11)
C5—C6—C7—C16	−0.77 (18)	C5A—C6A—C7A—C16A	−0.22 (16)
C16—C7—C8—C9	1.55 (19)	C16A—C7A—C8A—C9A	0.14 (17)
C6—C7—C8—C9	−178.76 (13)	C6A—C7A—C8A—C9A	179.50 (11)
C7—C8—C9—C10	−1.0 (2)	C7A—C8A—C9A—C10A	−0.13 (18)
C8—C9—C10—C11	−0.6 (2)	C8A—C9A—C10A—C11A	0.17 (18)
C9—C10—C11—C16	1.65 (19)	C9A—C10A—C11A—C16A	−0.23 (16)
C9—C10—C11—C12	−177.77 (12)	C9A—C10A—C11A—C12A	179.31 (11)
C10—C11—C12—C13	−178.69 (11)	C10A—C11A—C12A—C13A	−179.03 (10)
C16—C11—C12—C13	1.88 (16)	C16A—C11A—C12A—C13A	0.50 (16)
C11—C12—C13—C14	0.14 (17)	C11A—C12A—C13A—C14A	−0.75 (17)
C2—C1—C14—C15	−0.18 (15)	C2A—C1A—C14A—C15A	−1.18 (15)
C17—C1—C14—C15	178.83 (9)	C17A—C1A—C14A—C15A	175.69 (9)
C2—C1—C14—C13	−179.59 (10)	C2A—C1A—C14A—C13A	179.92 (10)
C17—C1—C14—C13	−0.58 (15)	C17A—C1A—C14A—C13A	−3.20 (16)
C12—C13—C14—C1	177.38 (10)	C12A—C13A—C14A—C1A	179.07 (10)
C12—C13—C14—C15	−2.04 (15)	C12A—C13A—C14A—C15A	0.15 (15)
C3—C4—C15—C16	177.55 (9)	C3A—C4A—C15A—C14A	0.21 (15)
C5—C4—C15—C16	−1.62 (15)	C5A—C4A—C15A—C14A	−179.68 (9)
C3—C4—C15—C14	−1.19 (15)	C3A—C4A—C15A—C16A	179.28 (9)
C5—C4—C15—C14	179.65 (9)	C5A—C4A—C15A—C16A	−0.60 (14)
C1—C14—C15—C16	−177.54 (9)	C1A—C14A—C15A—C4A	0.80 (15)
C13—C14—C15—C16	1.90 (14)	C13A—C14A—C15A—C4A	179.75 (9)
C1—C14—C15—C4	1.18 (14)	C1A—C14A—C15A—C16A	−178.27 (9)
C13—C14—C15—C4	−179.38 (9)	C13A—C14A—C15A—C16A	0.68 (14)
C10—C11—C16—C15	178.59 (10)	C10A—C11A—C16A—C7A	0.24 (15)
C12—C11—C16—C15	−1.96 (15)	C12A—C11A—C16A—C7A	−179.32 (9)
C10—C11—C16—C7	−1.12 (16)	C10A—C11A—C16A—C15A	179.89 (9)
C12—C11—C16—C7	178.33 (10)	C12A—C11A—C16A—C15A	0.34 (15)
C4—C15—C16—C11	−178.65 (9)	C8A—C7A—C16A—C11A	−0.19 (15)
C14—C15—C16—C11	0.08 (15)	C6A—C7A—C16A—C11A	−179.58 (9)
C4—C15—C16—C7	1.05 (15)	C8A—C7A—C16A—C15A	−179.84 (9)
C14—C15—C16—C7	179.78 (9)	C6A—C7A—C16A—C15A	0.77 (15)
C8—C7—C16—C11	−0.46 (16)	C4A—C15A—C16A—C11A	180.00 (9)
C6—C7—C16—C11	179.84 (10)	C14A—C15A—C16A—C11A	−0.92 (15)
C8—C7—C16—C15	179.83 (10)	C4A—C15A—C16A—C7A	−0.35 (15)
C6—C7—C16—C15	0.13 (16)	C14A—C15A—C16A—C7A	178.73 (9)
C2—C1—C17—O1	−3.46 (15)	C2A—C1A—C17A—O1A	−116.54 (12)
C14—C1—C17—O1	177.53 (9)	C14A—C1A—C17A—O1A	66.57 (14)

### Hydrogen-bond geometry (Å, °)

**Table:**

*D*—H···*A*	*D*—H	H···*A*	*D*···*A*	*D*—H···*A*
O1—H1···O1A^i^	0.84	1.87	2.6972 (12)	167
O1A—H1A···O1^ii^	0.84	1.89	2.7163 (12)	167

Symmetry codes: (i) −*x*+1, −*y*, −*z*+1; (ii) *x*, −*y*+1/2, *z*+1/2.
